# Smart Shockwave Responsive Titania-Based Nanoparticles for Cancer Treatment

**DOI:** 10.3390/pharmaceutics13091423

**Published:** 2021-09-08

**Authors:** Veronica Vighetto, Luisa Racca, Marta Canta, Joana C. Matos, Bianca Dumontel, Maria Clara Gonçalves, Valentina Cauda

**Affiliations:** 1 Department of Applied Science and Technology, Politecnico di Torino, C.so Duca Degli Abruzzi 24, 10129 Turin, Italy; veronica.vighetto@polito.it (V.V.); luisa.racca@polito.it (L.R.); marta.canta@polito.it (M.C.); bianca.dumontel@polito.it (B.D.); 2 Centro de Química Estrutural, Universidade de Lisboa, Av. Rovisco Pais, IST, 1000 Lisboa, Portugal; joana.matos@tecnico.ulisboa.pt (J.C.M.); clara.goncalves@tecnico.ulisboa.pt (M.C.G.); 3 Centro de Ciências e Tecnologias Nucleares, Instituto Superior Técnico, Universidade de Lisboa, 2685-066 Bobadela, Portugal; 4 Departamento de Engenharia Química, Instituto Superior Técnico, Universidade de Lisboa, Av. Rovisco Pais, 1000 Lisboa, Portugal

**Keywords:** amorphous titania nanoparticles, bovine serum albumin coating, shock waves, nanoparticles-assisted ultrasound

## Abstract

Nanomedicine is an emerging treatment approach for many cancers, characterized by having high sensitivity and selectivity for tumor cells and minimal toxic effects induced by the conventional chemotherapeutics. In these context, smart nanoparticles (NPs) are getting increasingly relevant in the development of new therapies. NPs with specific chemical composition and/or structure and being stimuli-responsive to magnetic, light or ultrasound waves are new promising tools. In the present work, amorphous-titania propyl-amine functionalized (a-TiO_2_-NH_2_) NPs, coated with bovine serum albumin (BSA), are stimulated with high energy shock waves to induce cytotoxic effects in cancer cells. First, a new method to coat a-TiO_2_-NH_2_ NPs with BSA (a-TiO_2_-NH_2_/BSA) was proposed, allowing for a high dispersion and colloidal stability in a cell culture media. The a-TiO_2_-NH_2_/BSA NPs showed no cancer cell cytotoxicity. In a second step, the use of shock waves to stimulate a-TiO_2_-NH_2_/BSA NPs, was evaluated and optimized. A systematic study was performed in in vitro cell culture aiming to impair the cancer cell viability: NP concentrations, time steps and single versus multiple shock waves treatments were studied. The obtained results highlighted the relevance of NPs design and administration time point with respect to the shock wave treatment and allow to hypothesize mechanical damages to cells.

## 1. Introduction

Cancer incidence and mortality are continuously growing worldwide. Only in 2020, 19.3 million new cases and 10 million cancer-related deaths were recorded. Cancer has become the second (or even first) leading cause of death (for people below 70 years old) in several developed countries, and its burden is expected to increase [1]. Furthermore, the health expenditures related to cancer care are steadily increasing and are further expected to grow drastically in the next years [2,3]. For these reasons, the research of new therapeutic strategies to fight cancer has raised great efforts in the last decades [4,5]. Conventional anticancer approaches, e.g., chemotherapy and radiotherapy, show lack of selectivity causing severe side effects, besides the extremely high costs [6,7]. With nanomedicine, new cancer therapies emerge, based on the use of nanomaterials for the diagnosis, treatment [8] and theranostic [9]. Their benefits are related to the nanometric size, enabling their direct interaction with the cell or sub-cellular compartment. Furthermore, nanomaterials loaded with drugs are used as nanocarriers in targeted and/or drug delivery systems [4,10], or directly cause cancer cells death due to their intrinsic cytotoxic properties. Recent advances propose the external stimulation of NPs by magnetic, light or ultrasound waves in order to: (i) trigger a cytotoxic response in close proximity to the cancer cells, or (ii) exploit a synergistic action between an external stimuli and the NPs (after NPs internalization) thus maximizing the NPs cytotoxic potential [8].

Titanium dioxide (titania, TiO_2_), a semiconductor approved by Food and Drug Administration (FDA), is characterized by its noteworthy photo-stability, photo-reactivity [11], and antibacterial properties [12], being its toxicity considerably less than other NPs with similar properties, such as zinc oxide or quantum dots [13]. Until now TiO_2_ NPs have been widely explored in anticancer therapies, both as drug delivery platforms and as cytotoxicity agents for cancer cells. TiO_2_ NPs have been employed as radiosensitizers in radiotherapy [14] and they are frequently reported in synergism with UV light or ultrasound (US) to produce reactive oxygen species (ROS) to perform photodynamic or sonodynamic therapies, respectively [8,14,15]. Recently, amorphous titania (a-TiO_2_) NPs have emerged as an effective alternative to the TiO_2_ crystalline polymorphs [16,17]. The a-TiO_2_ NPs can be an efficient ultrasound responding agents in sonodynamic therapy, because they could be photoactivated by sonoluminescent light and provoke mechanical damages to cells when cavitation bubbles collapse [18].

Since the exact mechanism of the NPs-assisted US therapy is still under debate, and in particular it is difficult to distinguish between thermal and non-thermal effects, the use of shock waves (SW) has been proposed. The objective is to reduce the thermal effects and focus on how non-thermal ones trigger the cytotoxicity, in combination with a second component, i.e., chemical compounds or nanoparticles [19,20]. Recently, some of us demonstrated the synergistic cytotoxicity of ZnO nanocrystals and SW in cervical cancer cells. The effects of single versus multiple SW treatments on cancer cells have been evaluated, demonstrating that three consecutive treatments in combination with such nanocrystals are necessary to achieve cancer cell death [20].

Despite the successful advancement, however, one of the biggest issues regarding the use of NPs for therapy and/or diagnosis stays on their stability in human biological fluids [21]. In the case of TiO_2_ NPs, its agglomeration reduces the photoactivity [22]. In addition, chemical/physical interactions may occur between NPs and body fluids biomolecules, forming a ‘protein corona’ at the NP surface [10,23] which may alter the NPs biological identity and their stability, biodistribution, toxicity, and ultimately their fate [4]. A typical strategy to overcome the corona formation is to envelope the NPs with an organic coating [24], namely phospholipids [18], polymers or proteins [21]. Albumin, a bio-compatible and non-immunogenic protein normally present in the human body [25], with a half-life of 19 days, represents a promising candidate to shield a-TiO_2_ NPs. Several albumin-related formulations have already been clinically approved, e.g., albumin-based Paclitaxel (Abraxane). Moreover, it is reported that albumin coating may improve the internalization in cancer cells [26]. Both human serum albumin (HAS) and bovine serum albumin (BSA) have been widely employed in nanomedicine, due to their similarity [25]. Examples of albumin-coated crystalline titania NPs have been reported in the literature, to optimize NPs monodispersity and their properties maintenance in biological media [27,28].

Here we investigate the effect of BSA coating on the colloidal stability of a-TiO_2_-NH_2_ NPs. This stabilization effect actually is of paramount importance to further evaluate the biological effect of a-TiO_2_-NH_2_/BSA NPs in cancer cells. In view of the potential therapeutic role of such a-TiO_2_-NH_2_/BSA NPs on hematological cancer, we also investigate the effect of BSA-coated NPs in citrated plasma and the absence of clotting.

Then we systematically study the optimization conditions for synergistic cytotoxic effect of shock waves (SW) and a-TiO_2_-NH_2_ /BSA NPs on cancer cells. We report the parameters that have to be taken into account, i.e., single versus multiple SW treatments, different NPs concentrations and different incubation conditions used to cell exposure. The obtained results suggest the potential role of mechanical damages induced by the a-TiO_2_-NH_2_/BSA NPs, which should be highly stabilized in cell culture media and free to oscillate under the acoustic stimulation. This study represents a first proof of concept, and more data should be collected to evaluate the translation to clinics, in terms of in vivo trials, application of the SW transducer to the skin and duration of treatments.

## 2. Materials and Methods

### 2.1. a-TiO_2_-NH_2_ NPs Synthesis

a-TiO_2_-NH_2_ NPs are synthesized through a novel, alkaline, and room temperature sol-gel protocol, based on a previously reported methodology [17]. Briefly, a volume (280 µL) of aqueous sodium silicate solution (SSS; Na_2_O·SiO_2_, 27% wt. % SiO_2_ from Sigma-Aldrich, Schnelldorf, Germany) as nucleating agent is diluted in 25 mL absolute ethanol (EtOH; 99.5% from Merck KGaA, Darmstadt, Germany) and placed under magnetic stirring for 15 min. A mixture of absolute ethanol and ammonium hydroxide (NH_4_OH, 25% *w*/*w* from Merck KGaA, Darmstadt, Germany) is added to the SSS suspension and left under magnetic stirring for an additional 15 min. After this time, the SSS suspension is placed in an ultrasound bath (TELSONIC, Bronschhofen, Switzerland, Tec-15, Economy-Cleaner) and 835 µL of titanium IV isopropoxide (TiPOT, Ti[OCH(CH_3_)_2_]_4_, 97% from Sigma-Aldrich, Schnelldorf, Germany) is quickly added, followed by 30 min of sonication. The (propyl-amine-) functionalization of a-TiO_2_ NPs is made in situ with propylamine groups through the methodology described above. After the 30 min of the suspension sonication, a volume of 3-aminopropyltriethoxysilane (APTES; H_2_N(CH_2_)_3_Si(OC_2_H_5_)_3_, 99%, purchased from Sigma-Aldrich, Schnelldorf, Germany) is added drop by drop (molar ratio of 8:2 of TiPOT to APTES). The mixture is then left under magnetic stirring for 24 h at room temperature. The final suspension is finally centrifuged (5591 rcf; 15 min) four times with bi-distilled water (conductivity 0–2 µS/cm^3^, pH 5.8–6.5).

### 2.2. a-TiO_2_-NH_2_ NPs BSA Coating

a-TiO_2_-NH_2_ NPs aqueous suspension is sonicated for 5 min. An aliquot of 100 μL is centrifuged at 10,000× *g* and re-dispersed in 10 mL of ultra-pure EtOH to obtain a 10 mg/mL ethanol suspension. The coupling with BSA (Bovine Serum Albumin from Sigma-Aldrich, Schnelldorf, Germany) starts from this 10 mg/mL ethanol suspension and is adapted from a previously described method [27,28]. The suspension is sonicated for 5 min and vortexed for 15 s. Another aliquot of 100 μL is diluted in 900 μL of sterile autoclaved MilliQ water to obtain a 1 mg/mL NPs aqueous suspension. The solution is vortexed 15 s, sonicated 15 min and vortexed again 15 s before further dilutions. Appropriate aliquots are diluted in 1 mL water/BSA solution (10 mg/mL) to obtain the desired concentration for the further characterizations.

### 2.3. NPs (a-TiO_2_-NH_2_, a-TiO_2_-NH_2_/BSA) Characterizations

X-Ray diffraction (XRD) patterns are collected by with a Panalytical X’Pert PRO diffractometer (Malvern, UK). Cu-Kα monochromatic radiation is used as the X-ray source with λ = 1.54059 Å. Field Emission Scanning Electron Microscopy (FESEM) imaging is performed with Zeiss Supra at 5 kV equipped by Energy Dispersive Spectroscopy (EDS) carried out at 15 kV and an acquisition time per pixel of 120 ms. In the above-mentioned characterization methods, the NPs suspension (prior and after BSA coating) in water media are deposited on silicon wafers and the suspensions let dry before recording XRD, FESEM or EDS analysis. Z potential (ζ) and dynamic light scattering (DLS) measurements are carried out with Zetasizer Nano ZS90 (Malvern). In particular, 100 µg/mL of pristine a-TiO_2_-NH_2_ and a-TiO_2_-NH_2_/BSA NPs are centrifuged at 5000× *g* for 5 min and re-suspended in 1 mL of bi-distilled water, or in 1 mL of cell culture medium RPMI-1640, or in 1 mL RPMI medium completed with 10% vol of fetal bovine serum (FBS), representing the real condition of cell culture in vitro, for the analyses. The Bradford assay is performed to quantify BSA absorption on NPs surface through the absorbance measurement at 590 nm with the Multiskan GO microplate UV–VIS spectrophotometer (Thermo Fisher Scientific, Waltham, MA, USA). After the generation of a calibration curve fitting known concentrations of BSA protein, the absorbances of triplicates from each sample (pristine a-TiO_2_-NH_2_ NPs and a-TiO_2_-NH_2_/BSA NPs at 50 µg/mL) are recorded.

### 2.4. Hemocompatibility

In order to evaluate the hemocompatibility of a-TiO_2_-NH_2_ and a-TiO_2_-NH_2_/BSA NPs, Human recovered plasma (from Zen Bio) was used in combination with calcium chloride (CaCl_2_ 0.025 M from HYPHEN BioMed SaS, Neuville-sur-Oise, France), as clotting agent. Using 96 well plate, 75 µL of plasma were plated for each sample, including controls (i.e., samples without CaCl_2_). Subsequently, 75 µL of a-TiO_2_-NH_2_ or a-TiO_2_-NH_2_/BSA NPs, with a concentration of 100 µg/mL in physiological solution, were added. As control, 75 µL of physiological solution only was added. Coagulation was started adding 75 µL of CaCl_2_.

The absorbance of each sample well was measured at 405 nm with a Multiskan GO microplate UV–VIS spectrophotometer (Thermo Fisher Scientific, Waltham, MA, USA), while maintaining the plate at constant temperature equal to 37 °C. Measurements were taken for 45 min, every 30 s as reported elsewhere [29]. Three replicates per sample were averaged to obtain the main absorbance at each time point and two independent experiments were conducted. The time corresponding to the half maximal absorbance (t_1/2_) was calculated for each profile. This value is related to the clot formation time because of the increase of plasma absorption during the clot formation, when the plasma becomes turbid, as previously reported [30].

### 2.5. Cell Lines and Reagents

The human Burkitt Lymphoma cell line Daudi (ATCC^®^ CCL-213TM) is obtained from the American Type Culture Collection (ATCC, Manassas, VA, USA). Daudi cells are cultured in ATCC-formulated RPMI-1640 Medium (ATCC30-2001) supplemented with 10% of heath inactivated fetal bovine serum (ATCC-302020), 100 units/mL penicillin and 100 µg/mL streptomycin (Sigma-Aldrich, Schnelldorf, Germany) and maintained at 37 °C under a 5% CO_2_ atmosphere.

### 2.6. Cytotoxicity Assay

Cell viability is determined with WST-1 cell proliferation assay (Roche, Basel, Switzerland) after 24 h treatment with scalar doses of a-TiO_2_-NH_2_/BSA NPs (from 10 to 100 µg/mL). The a-TiO_2_-NH_2_/BSA aqueous solutions (10–25–50–75–100 µg/mL) are centrifuged at 5000× *g* for 5 min. The supernatants are discarded, and the pellets are re-suspended in 1 mL of pre-warmed cell culture medium to obtain the final treatment suspensions. These suspensions are vortexed 15 s immediately before their addition to the cell culture. 2 × 10^4^ cells/well are plated in 100 μL/well with the treatment suspensions in 96 well flat bottom plates for suspension (Greiner-bio one, Kremsmünster, Austria) and incubated at 37 °C in 5% CO_2_. After 20 h, 10 µL of the WST-1 reagent are added to each well and after 4 h incubation at standard conditions, the WST-1 absorbance at 450 nm is detected by the Synergy H1 microplate reader (BioTek Instruments, Winooski, VT, USA) using a 620 nm reference. For the data analysis, the background signal of each treatment suspension is subtracted to the relative cell absorbance. Data are expressed as mean ± standard error mean and graphed with Origin (OriginLab). All experiments are performed at least in duplicates. Statistical analysis were performed with Origin (OriginLab).

### 2.7. Internalization and Fluorescence Microscopy

Internalization of a-TiO_2_-NH_2_/BSA NPs is evaluated by flow cytometry and fluorescence microscopy after 24 h and 48 h. For this assay, a-TiO_2_-NH_2_ NPs are first labelled overnight with Atto647-NHS, as previously described [18], and then coupled with BSA obtaining Atto647 TiO_2_-NH_2_-BSA NPs treatment suspension. Then, 2 × 10^5^ cells/mL are plated at 1 mL/well with the treatment suspension in 24 well flat bottom plates for suspension (Thermo Scientific, Waltham, MA, USA) and incubated at 37 °C in 5% CO_2_. For flow cytometry, after 24 h and 48 h incubations, the cells are washed twice with phosphate saline buffer (PBS, Sigma-Aldrich, Schnelldorf, Germany) through centrifugation at 130× *g* for 5 min, then re-suspended in 500 µL of PBS and analyzed with the Guava Easycyte 6-2 L instrument (Merck Millipore, KGaA, Darmstadt, Germany), as reported before [20,31]. For fluorescence microscopy, after 24 h and 48 h, cells are centrifuged (130× *g*; 5 min), resuspended in 50 µL of culture medium and spotted on a 4-well chamber slide (Thermo Scientific™ Nunc™ Lab-Tek™ II CC2™ Chamber Slide System, Waltham, MA, USA), pre-coated with polylysine (Sigma-Aldrich, Schnelldorf, Germany). After 30 min in the incubator, 450 µL of culture medium is added and cell membranes are labelled with 5 µg/mL of WGA-488 (Thermo Fisher Scientific, Waltham, MA, USA) for 10 min, and cell nuclei are stained with 0.4 µg/mL of Hoechst (Thermo Fisher Scientific, Waltham, MA, USA) for 5 min. Then, after three washing steps with live cell imaging suspension (LCI, Molecular Probes, Waltham, MA, USA), cells are re-suspended in 500 µL of LCI and imaged with a wide-field fluorescence-inverted microscope (Eclipse Ti-E, Nikon, Tokyo, Japan), equipped with an incubator gas chamber (Okolab) using an immersion oil 100× objective (Apo 1.40, Nikon, Tokyo, Japan).

### 2.8. Shock Waves Treatment

The cytotoxicity of SW alone, generated by PW^2^ device from Richard Wolf (Elvation Medical GmbH, Kieselbronn, Germany) is previously assessed. 2 × 10^4^ cells/well in 100 µL are seeded in 96 well plates for suspension and gradually exposed to SW treatment. 24 h later cell viability is recorded with the WST-1 assay. The combined effect of a-TiO_2_-NH_2_/BSA NPs and SW is then investigated evaluating different cytotoxic effects derived from NPs internalized/bound to cell membrane and non-internalized in Daudi cells further exposed to SW treatment. In the first case, Daudi cell are plated as for the internalization assay with different suspensions (culture medium, and culture medium with 25 and 50 µg/mL of a-TiO_2_-NH_2_/BSA NPs). 24 h later, cells are centrifuged (130× *g* for 5 min), counted and 2 × 10^4^ cells/well are plated in 100 µL in 96 well plates for suspension for a single or multiple (3 times/day, one treatment every 4 h) SW treatments. Otherwise, in the second case Daudi cells are plated in the 96 wells plate with the treatment suspensions (2 × 10^4^ cells/well in 100 µL) and immediately exposed to SW for single or multiple treatments as before. In both cases, the cell viability is recorded 24 h after the SW treatment with the WST-1 assay. Data are expressed as mean ± standard error mean and graphed with Origin (OriginLab v8.5). All experiments are performed at least in duplicates.

## 3. Results and Discussion

a-TiO_2_-NH_2_ NPs are synthesized at room temperature following an eco-friendly alkaline sol-gel route. Their morphological and structural characterization were already published [17,18]. In the present work, the a-TiO_2_-NH_2_ NPs are specifically coated with BSA protein to favor their colloidal dispersion to avoid aggregation in biological media, i.e., cell culture. The success of the coating step was confirmed by the Bradford assay. a-TiO_2_-NH_2_/BSA NPs reveals a BSA protein content of 177 µg/mL, as opposed to 0 µg/mL in case of a-TiO_2_-NH_2_ NPs.

**Figure 1:**
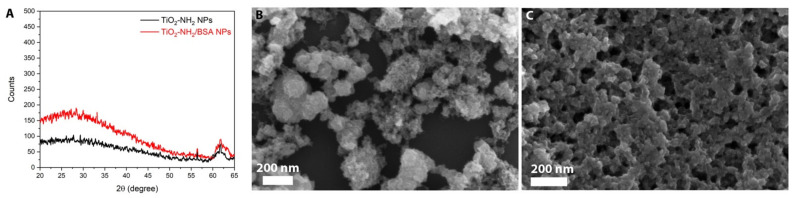
(**A**) X-ray diffraction pattern of aminopropyl-functionalized amorphous titania before (black curve) and after BSA coating (red curve); FESEM images of (**B**) a-TiO_2_-NH_2_ and (**C**) a-TiO_2_-NH_2_/BSA.

The X-Ray diffraction pattern in Figure 1A shows that the amorphous character of the a-TiO_2_-NH_2_ NPs is not modified by the BSA coating, as well as their morphological characteristics, as shown in Figure 1B,C by the FESEM images. The FESEM images (Figure 1B,C) represents tiny NPs aggregated in bigger agglomerates, which are partly due to both the imaging method, i.e., in high vacuum conditions, and the sample preparation method, see Materials and Method section.

**Figure 2:**
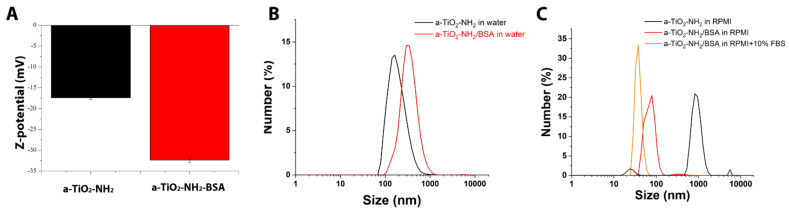
(**A**) Z-potential measurements in water of a-TiO_2_-NH_2_ (black bar and curves) and a-TiO_2_-NH_2_/BSA (red bar and curves); Dynamic light scattering measurements in number (%) (**B**) in water and (**C**) in cell culture medium RPMI and complete cell culture medium (for TiO_2_-NH_2_/BSA only, orange curve).

In order to study the effect of BSA-coating on colloidal stabilization, Z potential measurements and dynamic light scattering (DLS) are carried out (Figure 2). The Z potential measurements are performed with the NPs suspended in water, DLS with the NPs suspended in both water and cell culture media.

a-TiO_2_-NH_2_ NPs present a negative Z potential value (−17.4 mV), even with the presence of amine-groups that are typically protonated at pH ~7, in bi-distilled water (Figure 2C), in accordance with our previous results [18]. Although this Z potential value is an indirect proof of amine-functionalization, it also indicates a flocculation and/or coagulation risk associated with the a-TiO_2_-NH_2_ NPs (as Z potential value is within the ‘risk region’ |ζ| ≤ ±30 mV). Finally, BSA coating decreases Z potential value to −32.3 mV, which is outside the ‘risky region’ (|ζ| ≥ ±30 mV) [32].

Regarding the DLS analysis in water medium (Figure 2B), the average hydrodynamic diameter of the a-TiO_2_-NH_2_ NPs (black curve, peaking at 164 nm, PDI = 0.627) is smaller than the a-TiO_2_-NH_2_/BSA NPs (red curve, peaking at 295 nm, PDI = 0.308), as expected. Concerning the size distribution, a-TiO_2_-NH_2_/BSA NPs exhibit narrower peak values, when compared with a-TiO_2_-NH_2_ NPs ones. It is possible to observe an improvement on the suspension stability given by the BSA coating, not only in water (Figure 2B), as described above, but especially in cell culture medium (Figure 2C), when comparing the obtained results of the a-TiO_2_-NH_2_/BSA NPs with the uncoated a-TiO_2_-NH_2_ ones. A narrow size distribution is actually obtained when the a-TiO_2_-NH_2_/BSA NPs are in cell culture medium: peak at 78.8 nm (PDI = 0.404) in RPMI cell culture medium (red curve in Figure 2C) comparing with uncoated a-TiO_2_-NH_2_ particles. Heavy aggregation is observed in this last case, with micro-scale aggregates, broad and multiple peak size distribution and thus very poor dispersion.

To represent the real condition of cell culture, the cell culture medium is also completed with 10% vol with fetal bovine serum (FBS). In this case, a slight decrease and even narrower size distribution is observed down to 37.8 nm (PDI = 0.355) for a-TiO_2_-NH_2_/BSA NPs (Figure 2C, orange curve). Concerning the obtained results of the uncoated a-TiO_2_-NH_2_ NPs in the medium completed with 10% vol of FBS, these are not reported in Figure 2B due to the low quality of the data. In this case a heavy aggregation is observed with micro-scale aggregates and sample precipitation, which does not allow to collect the measurement properly.

The hemocompatibility of a-TiO_2_-NH_2_ and of a-TiO_2_-NH_2_/BSA was preliminary evaluated through a simple turbidimetric assay able to measure the kinetics of clot formation revealing that the plasma clotting time is not affected by the presence of both types of NPs, as shown in Figure S1 of the Supporting Information (S.I.). In particular, the time to reach half of the maximal absorbance, i.e., clot formation time, of plasma alone is 10.7 min and the ones obtained for plasma with the addition of a-TiO_2_-NH_2_ and of a-TiO_2_-NH_2_/BSA NPs are 9.4 min and 10.5 min, respectively. The absence of shift in the absorbance curve and the similarity of the times corresponding to half maximal absorbance values suggest that the presence of a-TiO_2_-NH_2_ and a-TiO_2_-NH_2_/BSA do not modify the physiological coagulation of plasma, suggesting a low interaction with blood coagulation, potential signal of good hemocompatibility of both NP formulations.

**Figure 3:**
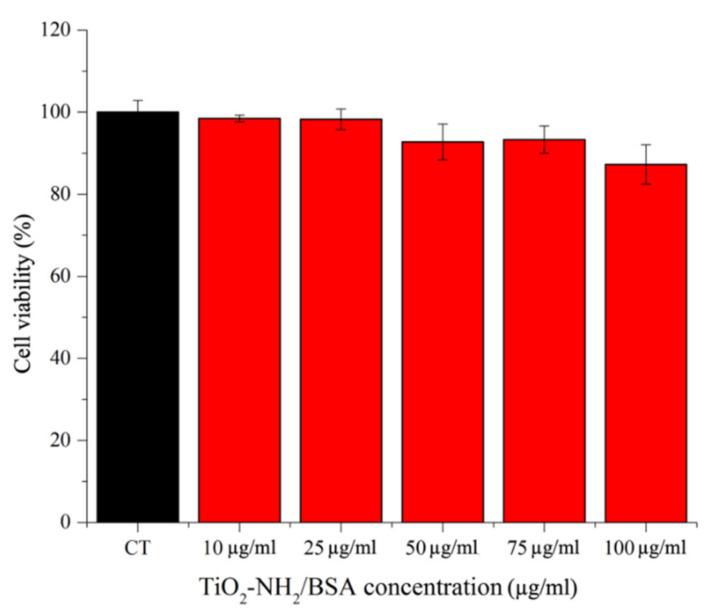
a-TiO_2_-NH_2_/BSA NPs cytotoxicity on Daudi cells incubated with different NPs concentrations (10, 25, 50, 75 and 100 µg/mL). Cell viability is measured after 24 h. Bars represent mean percentages of cell viability with respect to the control cells (CT) ± SEMs, *n* = 3.

After the preliminary characterization of a-TiO_2_-NH_2_/BSA core-shell nanostructure, the cell viability in a hematological cancer cell line, i.e., Burkitt lymphoma, is evaluated. A very good cell viability is recorded (Figure 3) up to 100 µg/mL, with a slight viability decrease from the safest concentration of 10 and 25 µg/mL to 50–75 µg/mL. The lowest viability value is recorded for 100 µg/mL which, however, is relatively high, with 90% of viable cells. It has to be pointed out that no cell viability experiments can be obtained from the pristine a-TiO_2_-NH_2_ NPs, since their heavy aggregation in cell culture medium (as observed above) prevents their use in in vitro cell culture.

**Figure 4:**
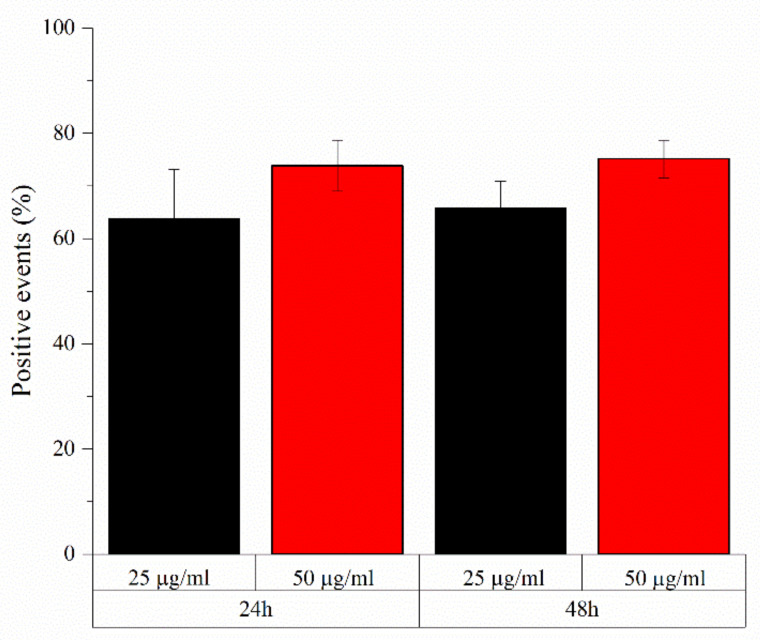
a-TiO_2_-NH_2_/BSA NPs internalization on Daudi cells incubated for 24 h and 48 h with different NPs concentrations (25 and 50 µg/mL). Bars represent the percentage of positive event with respect to the control cells ± SEMs, *n* = 3.

Similarly, a very high number of positive events, representing cells with a shift in fluorescence intensity with respect to control ones due to the presence of Atto647 labelled NPs which are internalized or attached to the cell surface, are recorded using 25 and 50 µg/mL of a-TiO_2_-NH_2_/BSA NPs after both 24 h and 48 h of co-incubation with such Daudi cancer cells, as shown in Figure 4. The highest positive events related to such core-shell nanostructures are recorded for the highest concentration used, i.e., 50 µg/mL, reaching 75.2% ± 2.9 of positive events after 48 h.

**Figure 5:**
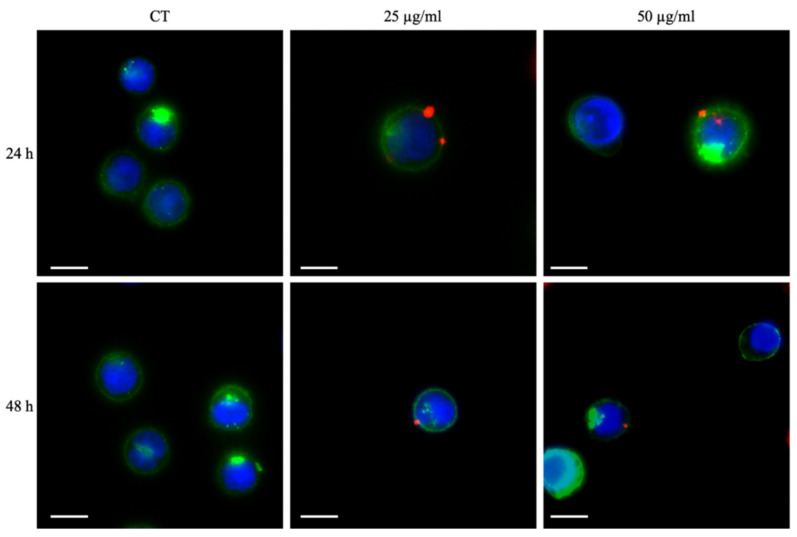
Fluorescence microscopy images of Daudi cells treated with different concentrations of TiO_2_-NH_2_/BSA NPs after 24 h and 48 h of incubation. Control cells without NPs (CT), cells incubated with 25 µg/mL and cells incubated with 50 µg/mL were analyzed. The blue channel represents the cells’ nuclei labelled with Hoechst, the green channel shows the cells’ membranes labelled with WGA488, and the red channel displays the NPs labelled with ATTO647. Scale bars are equal to 10 µm.

Fluorescence microscopy images in Figure 5 reports the comparison of the Daudi cells cultured with two concentrations of a-TiO_2_-NH_2_/BSA NPs (25 and 50 µg/mL) and after 24 h and 48 h. The cells membrane is labelled with green emitting dye while the cell nucleus with blue one. The a-TiO_2_-NH_2_/BSA NPs are depicted as tiny red spots and result clearly bound to the outer cell membrane surface, irrespectively with their concentration or incubation time.

In order to evaluate the effect of SW on Daudi cells, a preliminary characterization is performed in absence of the NPs to calibrate the number of shots and the correct time duration. An acceptable cell viability (>90%) is obtained by treating the Daudi cells with SW at 12.5 MPa of acoustic pressure and 250 shots, for 1 time per day or up to three times per day.

**Figure 6:**
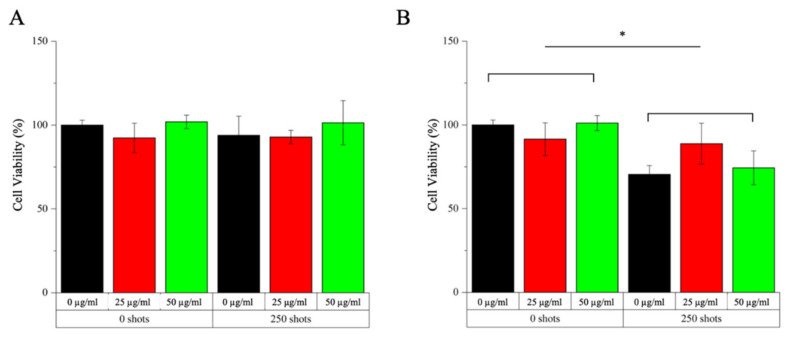
Study of INTERNAL effects with a-TiO_2_-NH_2_/BSA NPs on Daudi cell line in combination with single (**A**) and multiple (**B**) SW treatments (3 times/day). In each graph are depicted the conditions of cells in absence (0 shots) or treated with shock waves (250 shots). Different samples are prepared per assay: control untreated cells (0 µg/mL, black bars), cells incubated with 25 µg/mL a-TiO_2_-NH_2_/BSA NPs for 24 h (25 µg/mL, red bars), cells incubated with 50 µg/mL a-TiO_2_-NH_2_/BSA NPs for 24 h (50 µg/mL, green bars). Cell viability is recorded 24 h after the SW treatment with the WST-1 proliferation reagent. Data are reported as the cell viability with respect to the control referred as the 100%. Bars represent mean percentages of cell viability with respect to the control cells ± SEMs, *n* ≥ 2. 2-way ANOVA was performed to determine statistical significance (* *p* < 0.05).

**Figure 7:**
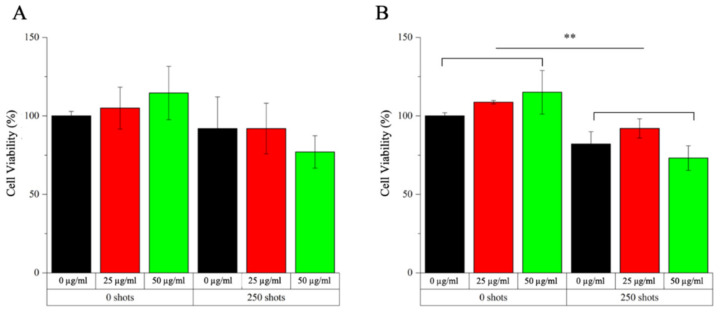
Study of EXTERNAL effects with a-TiO_2_-NH_2_/BSA NPs on Daudi cell line in combination with single (**A**) and multiple (**B**) SW treatments (3 times/day). Different samples are prepared per assay in absence (0 shots) or treated with shock waves (250 shots): control untreated cells (0 µg/mL, black bars), cells with 25 µg/mL a-TiO_2_-NH_2_/BSA in the cell culture medium (25 µg/mL, red bars) or cells with 50 µg/mL a-TiO_2_-NH_2_/BSA (50 µg/mL, green bars) and immediately treated with SW. Cell viability is then recorded 24 h after the SW treatment with the WST-1 proliferation reagent. Data are reported as the cell viability with respect to the control referred as the 100%. Bars represent mean percentages of cell viability with respect to the control cells ± SEMs, *n* ≥ 2. 2-way ANOVA was performed to determine statistical significance (** *p* < 0.01).

After these preliminary tests, studies combining safe doses of SW and NPs are conducted to explore possible enhanced cytotoxicity raising from the combination of the NPs with the external stimulation. The obtained results can be divided according to various tested conditions: (i) two different NPs concentrations, i.e., 25 and 50 µg/mL; (ii) presence or absence of shock waves at the maximum possible intensity and shot’s number (i.e., 12.5 MPa and 250 shots); (iii) number of SW treatments, i.e., single treatments (once a day) or multiple treatment (three times a day); (iv) NPs internalized/bound to the cell outer surface (i.e., after a co-incubation time of 24 h with cells–experiment called INTERNAL effects) or injected in the cell culture medium and immediately treated with SW (experiments called EXTERNAL effects). The results of these analysis are presented in Figure 6 for the INTERNAL effects and Figure 7 for the EXTERNAL ones.

Concerning the study of the internal effects no reduction of the cell viability is observed when cells are subjected to a single SW treatment, irrespectively to the concentration used of the BSA-coated titania NPs (Figure 6A). In comparison to these results, a decrease of cell viability is observed when applying the SW for three times/day (Figure 6B), already in absence of NPs. Furthermore, when applying multiple treatments after 24 h of incubation with the NPs at both concentrations, no additional cytotoxicity is recorded, thus any synergistic effects have to be excluded in this case.

The scenario is different when evaluating the EXTERNAL effects configuration, that is when the a-TiO_2_-NH_2_/BSA NPs are injected in the cell culture medium and then the system is immediately treated with shock waves once or three times. Actually, after a single treatment (Figure 7A) a reduction in cell viability is observed when applying SW with NPs at the concentration of 50 µg/mL with respect to the control bars. A further low cell viability is observed in Figure 7B, when applying multiple SW treatments and a-TiO_2_-NH_2_/BSA NPs at 50 µg/mL. In contrast the use of a lower NPs concentration, i.e., 25 µg/mL, does not seem to produce any synergistic effect in combination with SW able to induce cancer cell to death.

We also investigated the possible generation of ROS due to the application of shock waves to water and a-TiO_2_-NH_2_ NPs suspensions. As reported in Figure S2 of the S.I., no kind of ROS are detected by Electron Paramagnetic Resonance Spectroscopy neither in water nor in a-TiO_2_-NH_2_ NPs suspension (even at high concentrations, i.e., 200 µg/mL) when applying SW. Therefore, the potential cytotoxic effects caused by ROS generation in cell culture media must be ruled out in the present experiments.

From the above evaluations, it is clear, that a mechanical effect has to be considered when analyzing the viability of the obtained results in Figure 6 and Figure 7.

Furthermore, the administration method of the NPs to the cell culture is critical, since the highest levels of cytotoxicity are observed when the system is treated immediately after the NPs administration. In view of the cytofluorimetry and fluorescence microscopy results reported in Figure 4 and Figure 5, respectively, it is evident that this type of NPs bind to the outer cell membrane within the studied times, i.e., 24 and 48 h of co-incubation. Thus, it can be assumed that the NPs are somehow immobilized at the cell membrane surface. Possibly, the further application of shock waves is not able to displace the NPs from their position, inducing a limited number of mechanical damages to the cell. In contrast, when the NPs are injected into the cell culture medium and immediately subjected to SW, they can freely move and potentially produce mechanical damages to the cell membranes, as figuratively called here “nano-drill” effect. In this context, the use of repeated treatment (i.e., three SW stimulations per day every 4 h) instead of single ones is of paramount importance, preventing the cells to put in place efficient recovery mechanisms and inducing them to death.

## 4. Conclusions

In this work we establish a synergistic approach between SW and titania-based NPs, leading to cytotoxicity in cancer cells. We start by proposing a stabilization strategy through protein coating, i.e., adsorbing BSA protein on a-TiO_2_-NH_2_ NPs. These assemblages show a high colloidal stability in cell culture medium, allowing a direct interaction between them and the cancer cells.

Afterwards we independently explore the cell viability, incubating Daudi cancer cells at various a-TiO_2_-NH_2_/BSA NPs concentrations and exposing cancer cells to different shock waves stimulations, varying the acoustic pressure and the number of shots. After establishing the safest conditions for cells at the highest possible NPs concentrations and SW treatments, we propose their combination with the aim to induce cancer cell death. The results show that multiple shock wave treatments are preferable with respect to single one. Furthermore, the highest a-TiO_2_-NH_2_/BSA NPs concentration (50 µg/mL) is able to better cooperate with the SW therapy, producing the most effective cytotoxicity when NPs are simply incubated in the cell culture medium (a protocol here called EXTERNAL effect) without waiting for cell internalization. We thus propose a cytotoxic mechanism based on the so-called “nano-drill” effect, able to elicit mechanical damages on cells, possibly due to the free oscillation of the well-dispersed NPs in the cell culture medium when subjected to shock wave stimulation.

Further improvements of the proposed approach can be consolidated in the future, evaluating various multiple treatments, incorporating drug molecules into the proposed core-shell NPs, and enabling a site-selective treatment of hematological cancer cells, or even inducing a specific cell internalization using appropriate ligands at the nanoparticle surface.

## Supplementary Materials

The following are available online at https://www.mdpi.com/article/10.3390/pharmaceutics13091423/s1. Figure S1: Kinetic profile of plasma clotting monitoring the absorbance at 405 nm as a function of time. Figure S2: Spin-trapped Electron Paramagnetic Resonance spectroscopy analysis of the reactive oxygen species induced by shock waves in pure water and a-TiO_2_-NH_2_ NPs suspension in water.
