# Supplementary material for: Smart Shockwave Responsive Titania-Based Nanoparticles for Cancer Treatment
Source: Pharmaceutics. 2021 Sep 8;13(9):1423. doi: 10.3390/pharmaceutics13091423 (PMC8467828; doi:10.3390/pharmaceutics13091423)
Supplement: Supplementary file 1 [file pharmaceutics-13-01423-s001.zip › pharmaceutics-1349277-supplementary.pdf]

pharmaceutics

MDPI

Supplementary Materials: Smart shockwave responsive titania-based nanoparticles for cancer treatment

Veronica Vighetto, Luisa Racca, Marta Canta, Joana C. Matos, Bianca Dumontel, M. Clara Gonçalves and Valentina Cauda

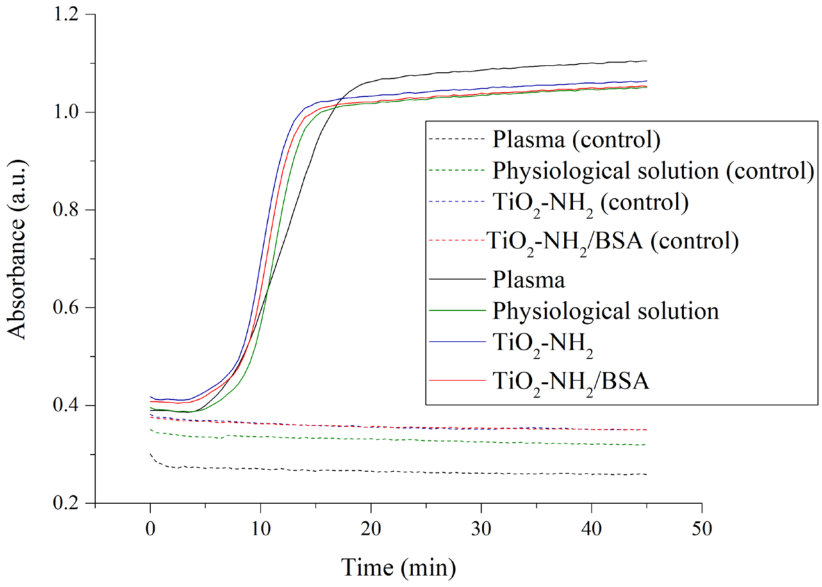

Figure S1. Kinetic profile of absorbance at 405 nm as a function of time. Dotted lines represent samples without the addition of the clotting agent; they are negative controls. Data show the mean in time of three replicates for each sample.

The evaluation of ROS generation under the stimulation with shockwaves (SW) was performed by Electron Paramagnetic Resonance (EPR) Spectroscopy through the EMX Nano X-Band Spectrometer from Bruker (Billerica, Massachusetts, US). The EPR measurements were assisted by a spin-trapping technique and the ROS production due to SW stimulation was provided by a PW² device from Richard Wolf. The sonication was performed by placing 100 µl of water or 100 µl sample (200 µg/mL concentration of a-TiO_2-NH_2 NPs in water) in a 96-well plate for suspension from Thermo Scientific, which was positioned in contact with the PW² transducer using an ecographic water-based gel (Stosswellen Gel Bestelle from ELvation Medical GmbH, Kieselbronn, Germany) to guarantee the contact. The conditions used for all samples were the same used to treat cells, as reported in the main paper, i.e., 12.5 MPa of acoustic pressure and 250 shots. After the US stimulation, the sample was immediately transferred into a quartz microcapillary tube and placed in the EPR spectrometer cavity. The production of hydroxyl and superoxide anion radicals was detected in bidistilled water and 5,5-dimethyl-L-pyrroline-*N*-oxide (DMPO, from Sigma-Aldrich, Schnelldorf Germany), was used as a spin trap. Before each measurement, DMPO (from a stock solution of 10 mM) was added to each tested sample. The spin trap is essential due to its ability to trap both radicals (hydroxyl and superoxide anion), increasing their lifetime and enabling their measurement by the EPR spectroscopy. The parameters to collect the EPR spectra were the following: center field 3426 G, sweep

time 100.0 s, sample g-factor 2.000, and number of scans 10. After the acquisition, the spectra were processed through the Bruker Xenon bidistilled software v2 for baseline correction. Analysis of the recorded data was made using the Bruker SpinFit software.

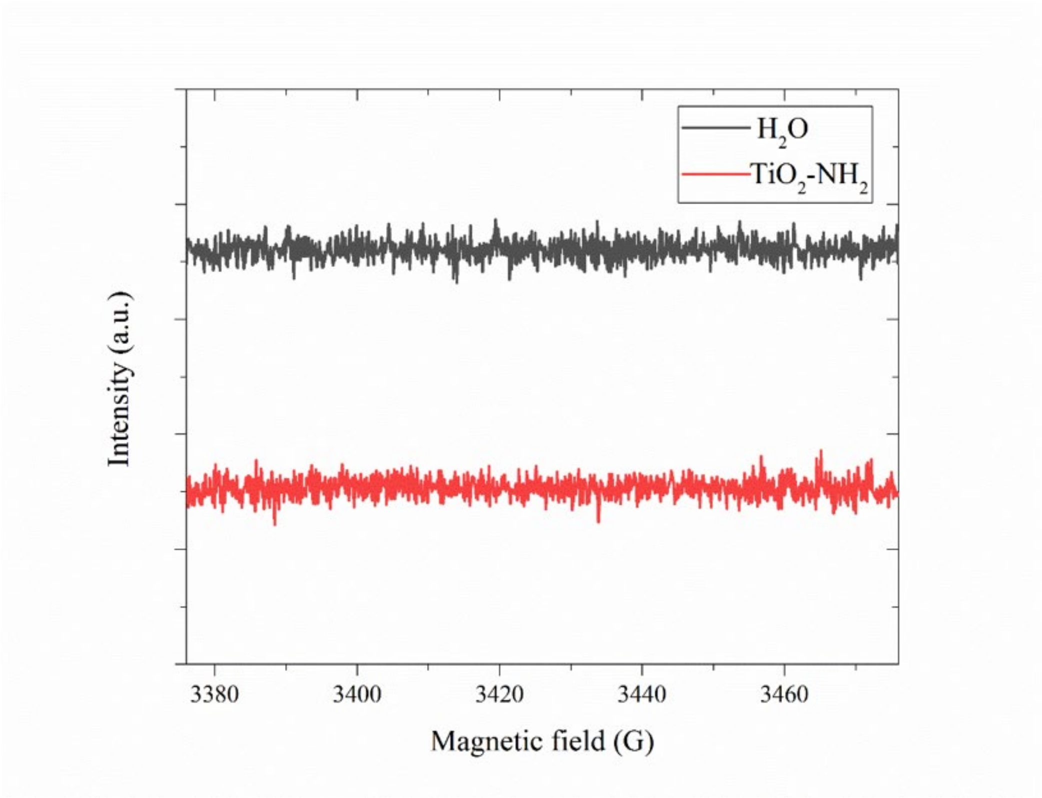

Figure S2. Spin-trapped Electron Paramagnetic Resonance spectroscopy analysis of the reactive oxygen species induced by shockwaves in pure water and water suspension.
